# Embedding evidence of early postoperative off-bed activities and rehabilitation in a real clinical setting in China: an interrupted time-series study

**DOI:** 10.1186/s12912-022-00883-5

**Published:** 2022-04-27

**Authors:** Yun Chen, Jing Wan, Zheng Zhu, Chunhong Su, Zhengrong Mei

**Affiliations:** 1 Department of Obstetrics and Gynecology,Guangdong Provincial Key Laboratory of Major Obstetric Diseases, The Third Affiliated Hospital of Guangzhou Medical University, Guangzhou, China; 2 School of Nursing, Fudan University, Shanghai, China

**Keywords:** Implementation science, Caesarean section, Postoperative, Early mobilization, Nursing

## Abstract

**Background:**

Patients should be encouraged to mobilize with 24 h of caesarean section. However, the time of the first off-bed activity after surgery is usually 24 ~ 48 h in China. Due to the lack of knowledge of early off-bed activities, lack of attention to medical pain, and the absence of systematic evidence for the clinical transformation process. the aim of this study was showed that the application of evidence needs to be embedding in the real setting to construct the localization plan and achieve the effective result.

**Methods:**

To establish evidence of the benefits of early postoperative off-bed activities on patients’ well-being based on a literature review. An interrupted time series analysis was used to evaluate the effectiveness of the intervention. The first and third periods were both five months (from February 1st, 2019 to January 31st, 2020), with a two-month interrupted time (from July 1st, 2019 to August 31st, 2019).

**Results:**

Eight clinical practices were retrieved from the literature and incorporated into the intervention. A total of 465 patients were included: 226 patients before and 239 patients after implementing the intervention. The average onset time of postoperative off-bed activities was significantly earlier after the intervention than before the intervention (20.01 vs. 31.89 h after the operation, *P* < 0.001). The 24-h off-bed rate increased from 30.94% before to 91.21% after the intervention (*P* < 0.001). The average pain score of patients decreased from 5.23 points before to 3.82 points after the intervention (*P* = 0.032). The average postoperative hospital stay was shortened from 5.06 days before to 3.51 days after the intervention (*P* < 0.001). In addition, the incidence rates of postoperative ileus (POI) and infection decreased from 5.38% and 2.65% before to 1.67% and 0.84% after the intervention, respectively (*P* < 0.001).

**Conclusions:**

We established an evidence-based nursing intervention. Evaluation of the effect of evidence-based practices should be considered in the clinical setting and include preoperative health education, effective analgesia management, and safety management.

**Supplementary Information:**

The online version contains supplementary material available at 10.1186/s12912-022-00883-5.

## Background

A caesarean section (C-section) is the only safe option to deliver the baby of a woman experiencing serious complications such as dystocia [1]. C-section is a widely used practice in clinical gynaecology and obstetrics [2, 3].

The rate of C-sections in developed countries remains high and continues to show an upwards trend [4]. The C-section is also quite common in China [5]. After the implementation of the two-child policy in this country, the incidence of placenta accreta has increased dramatically in recent years [6]. At present, caesarean section is the main option for the termination of pregnancy in a case of abnormally implanted placenta, which is invasive and traumatic, making postoperative recovery slow and difficult [6]. Consequently, post caesarean care is becoming increasingly significant and challenging for obstetrics care centres in China [7].

Due to the invasive nature of C-section deliveries, postoperative recovery is slow and usually requires 3–4 days in the hospital [6]. Evidence suggests that early mobilization can reduce the risk of complications during puerperium and enhance the safety and health of mothers and babies [7]. However, there is a knowledge gap among institutions, and adherence to the evidence supporting early mobilization remains highly variable [8–10]. The acceptance of Enhanced Recovery After Surgery (ERAS) has been slow in the field of emergency medicine, but recent research has found that ERAS utilization in the emergency setting is possible and effective, but certain changes to the protocol may need to be adapted [8, 9]. To implement evidence-based practices successfully, health care providers should tackle existing barriers [9]. Since facilitators and barriers are dependent on context, it is important to examine them in the clinical setting for which the protocol is intended and to supply the protocol of structured postoperative mobilization [10].

This study aims to develop tailored implementation strategies that optimise and standardise preoperative care of the early mobilization of patients after C-section and embed them in the clinical setting. Five guidelines [11–15] were retrieved via systematic review, and eight recommendations were developed based on the Grades of Recommendation, Assessment, Development, and Evaluation framework. We embedded this evidence into a clinical setting in China and evaluated the effectiveness of the length of postoperative hospitalization stay and the incidence rates of postoperative ileus (POI) and infection.

## Methods

We conducted an interrupted time-series study of early mobilization after C-section based on a literature review in the Guangzhou Obstetrics Critical Care Center from February 1st, 2019, to January 31st, 2020. This study was approved by the clinical scientific research section of the Ethics Committee of Guangzhou Medical University. Informed consent was obtained from every participant in this study.

### Literature review

A systematic literature search to identify all publications (research articles, practice guidelines, etc.) relevant to post caesarean care for rapid rehabilitation was conducted on the most relevant databases/websites, including Pub Med, British Medical Journal (BMJ) Best Practice, Elsevier Science Direct, Evidence-based Health Care Database (Joanna Briggs Institute, JBI), National Institute for Clinical Excellence (NICE), National Guideline Clearinghouse (NGC), Chinese Society of Obstetrics and Gynaecology (CSOG), Chinese Society of Surgery (CSS), Enhanced Recovery After Surgery (ERAS) Society, Chinese Biomedical Literature Database, Fudan Evidence-based Nursing Center Practice Guide (FENCPG), Wan-Fang Database, and China National Knowledge Internet (CNKI). The search was conducted using various combinations of subject words and free words. The English keywords included ‘early rehabilitation, early recovery, speed up rehabilitation, enhanced recovery, and fast track survey’. Keywords included ‘off-bed in early-stage, rehabilitation in early-stage, rapid rehabilitation, accelerated rehabilitation, and activities in the early stage’. All reports published from database/website creation to August 1st, 2019, were retrieved. Reports published publicly, available in full text, and written in either Chinese or English were included for further analysis. Studies on newborns, children, men, or patients undergoing non abdominal surgery were excluded. The literature evaluation was independently conducted by two researchers with expertise in evidence-based nursing. If the two reviewers could not agree on inclusion of a report, a postgraduate tutor was consulted.

### Clinical intervention

The guidelines were implemented in an interrupted time series (ITS) study. The patients were selected using the cluster sampling method. A total of 456 patients who underwent C-section deliveries in the Obstetrics Critical Care Center of Guangzhou from February 1st, 2019, to January 31st, 2020, were selected.

Inclusion criteria were as follows: patients who were admitted to the hospital and remained for more than 24 h and delivered via C-section, including emergency and elective surgeries.

Exclusion criteria were as follows: patients who were required to stay in bed for more than 24 h or were transferred to the intensive care unit (ICU) after the operation.

Collaboration: all of the stakeholders included hospital managers, obstetricians, nurses, anaesthesiologists, pharmacists, patients and caregivers.

Research setting: The Guangzhou Obstetrics Critical Care Center is the first severe maternal care centre in southern China and was established in 1998 to treat patients from throughout the country. There are 47 inpatient beds with multidisciplinary teams providing emergency services in cases including abnormally implanted placenta, preeclampsia, and amniotic fluid embolism. The proportions of high-risk pregnancies and urgent/critical pregnancies among hospitalized patients were 75% and 40%, respectively. Nursing care is provided by registered nurses. At the time of this study, 20 full-time registered nurses were employed.

### Implementation procedures

#### Preoperative patient education

In addition to the one-on-one bedside education that nurses practised before implementation of the intervention, new forms of health education were introduced, including a regular broadcast of educational videos on TV, presentation of educational materials in the corridor of the ward, distribution of informative manuals to patients, and regular educational lectures for family members. The educational materials included information about the clinical significance of early postoperative off-bed activities, forms of suitable off-bed activities, daily activity objectives, activity recording methods, pain management schemes, and early drainage tube withdrawal. Preoperative patient education, which took 15 to 20 min, was provided within 24 h after admission and when nurses performed preoperative patient care according to the doctor’s advice.

#### Pain management

A multimodal analgesia scheme for pain management during and after the operation was developed by directors and medical team leaders of the Department of Anaesthesiology and Pharmacy according to the hospital resources and patients’ preferences. The analgesia scheme included personalized analgesic drug delivery during surgery, such as the transversus abdominis plane (TAP) block, local infiltration anaesthesia before abdomen closure, and postoperative low-dose intraspinal or intravenous anaesthesia (for up to 48 h). Postoperative pain was regularly evaluated by anaesthesiologists, pharmacists, and clinical nurses, and analgesic drugs were administered according to the visual analogue scale (VAS) score [16]. The type and dosing of the analgesic drugs were adjusted to maintain a pain score below 3 [16] without causing safety concerns for the mother and baby.

#### Early extubation

After the surgery, the drainage tube in the pelvic cavity and the urinary catheter were evaluated by an experienced doctor twice daily and withdrawn as soon as possible.

#### Safety management

The patients had their first off-bed activities in the presence of a nurse, and their safety, including mental status, pain level, and tube/catheter status, was also assessed. Before getting out of bed with the help of nurses or family members, the patient practised sitting up for 30 s, positioning the foot perpendicular to the lower leg for another 30 s, and then stood for 30 s [7].

### Data collection

The investigation was performed in three time periods. The first period was a five-month (February 1st, 2019 to June 30th, 2019) baseline survey. The second period was a two-month (July 1st, 2019 to August 31st, 2019) transition to implement the intervention, including preoperative patient education and postoperative pain and safety management. The third period was a five-month (September 1st, 2019 to January 31st, 2020) evaluation of the effects of the intervention. The intervention was introduced to participants from July 1st, 2019, to January 31st, 2020. Patient outcomes were recorded continuously throughout the 12-month investigation by the deputy chief nurse and a nurse practitioner, and the data were analysed using the ITS method. Specifically, the autoregressive integrated moving average (ARIMA) model was used to compare patient outcomes before and after the intervention.

There were 10 core members on the project team, and they were all from the Obstetrics Critical Care Center. The director and the head nurse of the centre were responsible for the overall planning and execution of the project. The director of the anaesthesia department and the deputy chief pharmacist of the clinical pharmacy department were responsible for clinical supervision and consultation. The deputy chief nurse and a nurse practitioner were responsible for formulating the evidence-based intervention, personnel training, data collection, and analysis. A graduate supervisor was responsible for scientific supervision and consultation. The other core members of the project team were the medical and nursing team leaders of clinical treatment groups responsible for the project’s clinical execution.

### Measurement

A quality control team was formed comprising selected core members of the project team. All of the doctors and nurses received professional training on postoperative care for early off-bed activities. Members of the quality control team visited preoperative patients once or twice daily to ensure the accurate implementation of various intervention measures. A database comprising all patients’ data was updated monthly by two nurses in charge of bedside education.

#### Demographic and clinical characteristics

Demographic and clinical characteristics were collected by a standard demographic questionnaire. Demographic variables included age and education level (technical secondary school or less, junior college, college and master’s degree or more). Clinical variables included gestational age (weeks), type of operation (emergency or elective), diagnosis of high-risk pregnancy (scarred uterus, placenta previa/placenta accreta, pregnancy complicated by hypertensive disorders), duration of operation (hours), and intraoperative blood loss (ml). Clinical variables were verified by clinical nurses based on the patients' medical records.

#### Implementation of evidence-base practices

The head nurse evaluated the overall evidence-based practices of the intervention in a patient through bedside observation, patient interview, and review of the patient’s medical records. The results were recorded as ‘Y’ for meeting the standards, ‘N’ for failing to meet the standards and ‘NA’ for not applicable. The percentage of Y for each measure was calculated as an indicator of the effectiveness of implementation. The time of tube/catheter insertion and removal, pain evaluation and management were verified by the deputy chief nurse on the project team based on the patient’s medical records. The recovery time of intestinal peristalsis (anal exhaust time) after surgery and the time of the patient's first ambulation were also recorded.

#### Complications after C-section

The medical team leader assessed whether the patient had POI [17] or postoperative infection [13], in accordance with the diagnostic criteria for POI and postoperative infection, incidence of complications (POI or postoperative infection) = number of complications (POI or postoperative infection)/number of C-sections × 100%.

### Statistical analysis

Statistical analysis was performed with SPSS 21.0 software (IBM Corp., Armonk, NY, USA). Continuous data with normal distribution are presented as the means ± standard deviations (SD), and they were interpreted with the independent Student’s *t test*. Non normally distributed data are presented as the mean (min, max), and they were analysed with the Mann–Whitney U test. Categorical data are presented as frequencies (percentages). Differences between the two groups were interpreted using the chi-square test. A *P* value less than 0.05 was considered statistically significant.

## Results

### Review of the evidence

**Fig. 1:**
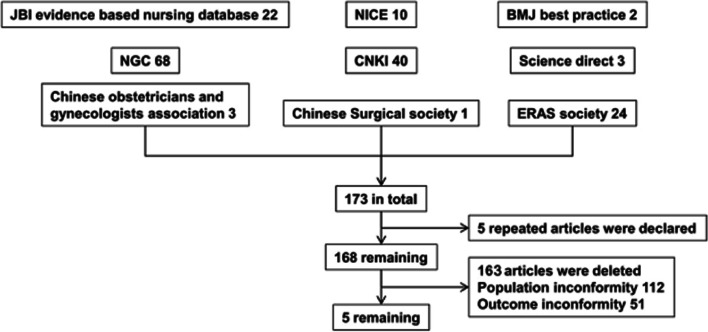
Flow chart of evidence retrieval

As shown in Fig. 1, a total of 173 relevant publications were retrieved, which included 68 from NGC, 10 from NICE, two from BMJ Best Practice, three from Elsevier Science Direct, 22 from FENCPG, 40 from CNKI, 24 from ERAS Society, three from CSOG, and one from CSS. The literature quality evaluation standard [18] developed by JBI Australia was used to evaluate the literature, and reports with a score of less than 70% were excluded. A total of 168 articles were excluded. They were five duplicate articles, 112 that did not encompass the study population, and 51 that were not in line with this study’s research outcomes. The five articles that passed the quality evaluation test, including one from JBI, one from PubMed, one from ERAS Society, one from CSOG, and one from CSS, were used as the sources of evidence for establishing the intervention. They were one evidence summary [11], two clinical guidelines [12, 13], and two expert consensuses [14, 15]. Finally, eight clinical practices were retrieved from these articles and incorporated into the intervention (Table 1).

**Table 1:** The clinical practices incorporated into the evidence-based intervention for promoting early postoperative off-bed activities

Clinical practices	Evidence	Evaluation indicators	Methods
Preoperative education	The health notice of early postoperative activities should be provided to patients; the postoperative activity plan with daily activity goals should be formulated and communicated to patients (Grade V)	Patients’ awareness of postoperative activity plan before the operation	Questionnaire Survey
		Patients’ awareness of daily activity goals before the operation	
Catheter management	The abdominal drainage tube should be avoided, and should be removed as soon as possible if it is used (Grade I)	Decreased use of indwelling abdominal drainage tube	Field observation
		Removal of the abdominal drainage tube within 24 h after the operation	Medical record viewing
	The urinary catheter should be removed within 24 h after the operation (except for patients with bladder repair) (Grade I)	Removal of the urinary catheter within 24 h after the operation	Field observation, Medical record viewing
Pain management	The best pain management plan for promoting early postoperative activities should be provided to patients (Grade I)	Regular pain assessment by medical staff	Questionnaire Survey
		The pain score kept below 3 points	Medical record viewing
	The use of opioids should be avoided if possible to allow early postoperative activities (Grade I)	Reduced use of opioid analgesics	Medical record viewing
Postoperative guidance	Off-bed within 24 h after the operation should be guided (Grade I)	Off-bed activities guided by nurses within 24 h after the operation	Field observation

### Participants in the intervention

**Fig. 2:**
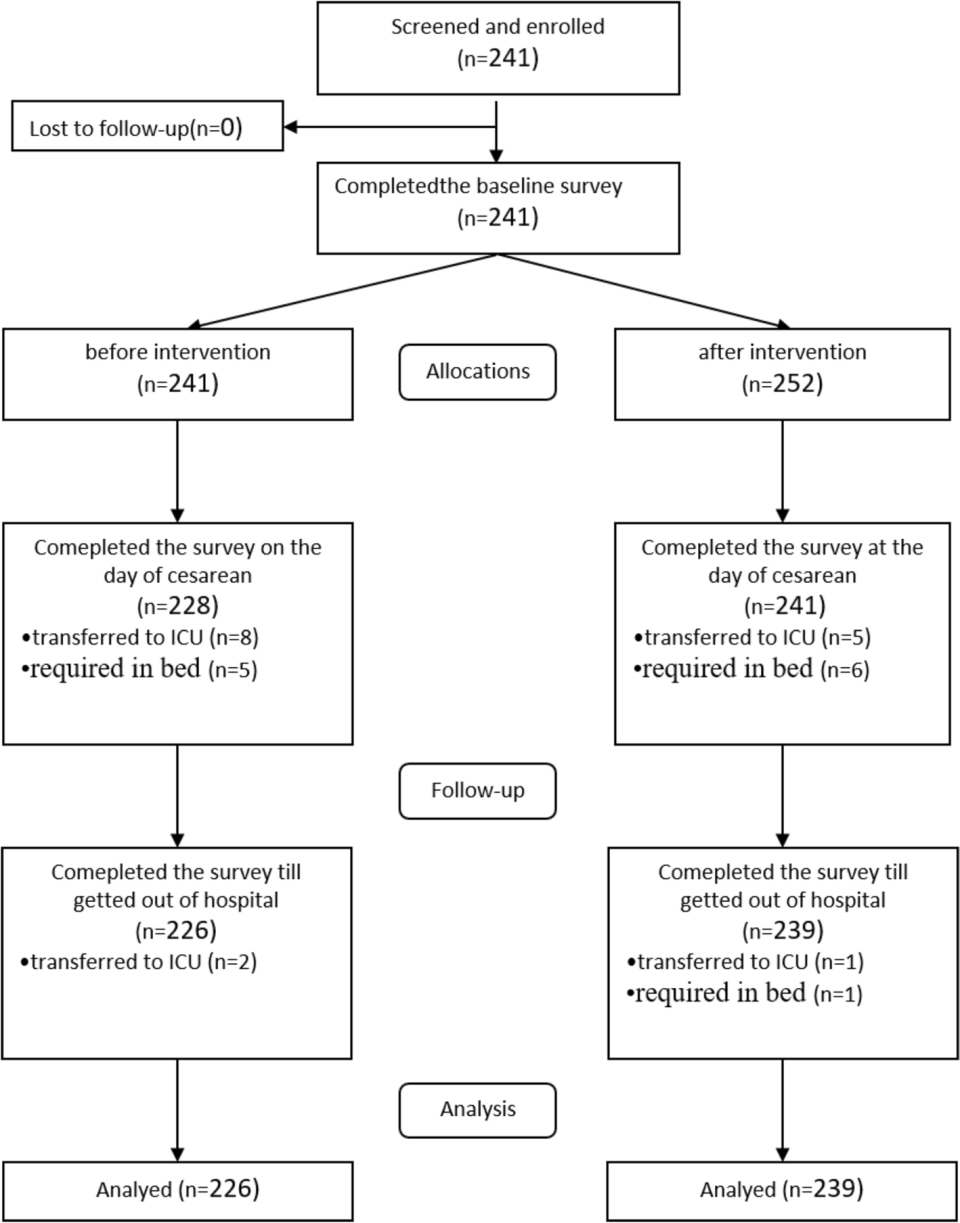
Flow chart of participants

This study included 241 participants who underwent C-sections before implementation of the evidence-based intervention (February 2019 to June 2019) and 252 participants who had C-sections after the intervention (September 2019 to January 2020). Fifteen participants before the intervention and thirteen participants after the intervention dropped out of the study before the final survey (Fig. 2). The demographic and clinical characteristics of patients before and after the evidence-based intervention are presented in Table 2. A total of 226 patients were included before the intervention. The patients had an average age of 32.25 ± 5.45 years, their babies had an average gestational age of 34.74 ± 3.24 weeks, average C-section operation time was 1.31 ± 0.63 h, and average intra-operative blood loss was 527 (434–774) mL. A total of 239 patients were enrolled after the intervention. These patients had an average age of 32.53 ± 5.30 years, average gestational age of 35.57 ± 3.65 weeks, average C-section operation time of 1.28 ± 0.56 h, and average intraoperative blood loss of 463 (378–615) mL. There were no statistically significant differences in patients’ ages, babies’ gestational ages, type of operation, type of anaesthesia, duration of operation, intraoperative blood loss, or preoperative diagnoses of high-risk pregnancies between the two patient groups.

**Table 2:** The demographic and clinical characteristics of patients before and after the evidence-based intervention

Characteristics		Before the intervention	After the intervention	*P* value
		(*n* = 226)	(*n* = 239)	
Age (years, mean ± SD)		32.25 ± 5.45	32.53 ± 5.30	0.236
Education level, N (%)	Technical secondary school or less	106 (46.90)	110 (46.03)	0.368
	Junior college	41 (18.14)	46 (19.25)	
	College	71 (31.42)	77 (32.21)	
	Master’s degree or more	8 (3.54)	6 (2.51)	
Gestational age (weeks, mean ± SD)		34.74 ± 3.24	35.57 ± 3.65	0.325
Type of operation, N (%)	Emergency operation	107 (47.35)	109 (45.61)	0.987
	Elective operation	119 (52.65)	130 (54.39)	
Diagnosis of high risk pregnancy, N (%)	Scarred uterus	103 (45.58)	107 (44.58)	0.235
	Placenta previa/placenta accreta	82 (36.28)	91 (38.08)	
	Pregnancy complicated by hypertensive disorders	76 (33.63)	77 (32.21)	
	Other complications of pregnancy	31 (13.72)	42 (17.57)	
	Pregnancy complicated by internal and external diseases	32 (14.29)	29 (12.13)	
Duration of Operation (h, mean ± SD)		1.31 ± 0.63	1.28 ± 0.56	0.562
Intra-operative blood loss (mL)		484 (382–714)	463 (378–615)	0.257
Exceeding 500 ml, N (%)		169(74.78)	177(74.06)	0.453
Type of anaesthesia, N (%)	Combined spinal-epidural anaesthesia	143 (63.27)	153 (64.02)	0.189
	General anaesthesia with intravenous inhalation	74 (32.74)	81 (33.89)	
	Other types of anaesthesia	9 (3.98)	5 (2.09)	
Type of operation, N (%)	Low-segment C-section	173 (76.55)	178 (74.48)	0.337
	Corporeal C-section	53 (23.45)	61 (25.52)	
	Ascending ligation of uterine artery	58 (25.66)	64 (26.77)	
	Pelvic adhesiolysis	82 (36.28)	84 (35.15)	
	Uterus repair	29 (12.83)	30 (12.55)	
	Subtotal hysterectomy	14 (6.19)	15 (6.28)	
	Bladder repair	8 (3.54)	8 (3.35)	

*SD* standard deviation

### Implementation rate of evidence

Before the implementation of the evidence-based intervention, the compliance rates of patients regarding the postoperative activity plan and daily activity goals were 0%, the compliance rate of reduced postoperative use of opioids was approximately 15%, and the compliance rates of other evaluation indicators varied from 45.7% to 84.5% (Table 3). After implementing the evidence-based intervention, the compliance rates of all evaluation indicators except two were over 80% and were significantly higher than those observed before the intervention (*P* < 0.05, Table 3). The two exceptions were abdominal drainage tube removal within 24 h after the operation and activities recorded by patients with a ground scale, which showed similar poor compliance levels before and after the intervention (*P* > 0.05, Table 3).

**Table 3:** Compliance rates of evaluation indicators before and after the evidence-based intervention

Evaluation indicators	Before the intervention (*n* = 226)				After the intervention (*n* = 239)				P
	Implemented (Cases)	Not implemented (Cases)	Inconformity (Cases)	Implementation rate (%)	Implemented (Cases)	Not implemented (Cases)	Inconformity (Cases)	Implementation rate (%)	
1. The patient was informed of the activity plan in written form before the operation	0	226	0	0	198	41	0	82.88	< 0.001
2. The patient was informed of the daily activity goals in written form before the operation	0	226	0	0	210	29	0	87.87	< 0.001
3. The retention rate of abdominal drainage tube during the operation was reduced	191	35	0	84.51	222	17	0	92.89	< 0.001
4. The abdominal drainage tube was removed within 24 h after the operation	16	19	191	45.71	7	10	222	41.17	0.73
5. The indwelling catheter was removed within 24 h after the operation	132	87	7	60.27	225	6	8	97.40	< 0.001
6. The pain was regularly assessed by the medical staff	168	58	0	74.34	234	5	0	97.91	< 0.001
7. The pain was controlled below a score of 3 points	168	58	0	74.34	200	39	0	86.88	0.04
8. The postoperative use of opioids was reduced	8	44	174	15.38	36	6	194	86.66	< 0.001
9. The off-bed activities within 24 h after operation were guided by nurses	148	78	0	65.49	226	13	0	94.56	< 0.001
10. The first off-bed activities were guided by nurses step by step	107	119	0	47.34	222	17	0	92.89	< 0.001
11. The patients’ off-bed activities were assessed by nurses on a daily basis	117	109	0	51.76	192	47	0	80.33	< 0.001
12. The patients recorded activities with a ground scale	108	118	0	47.79	143	96	0	59.83	0.46

### Postoperative rehabilitation of patients

Compared with before the evidence-based intervention, the onset time of postoperative off-bed activities after the intervention was significantly earlier (20.01 h vs. 31.89 h after the operation, *P* < 0.001, Table 4). The length of postoperative hospital stay decreased from 5.06 days before to 3.51 days after the intervention (*P* < 0.001, Table 4). The rate of successful postoperative pain control (pain score < 3) increased from 74.37% before to 83.68% after the intervention (*P* = 0.014, Table 4). It is worth noting that the average pain score of patients in the active state showed significant improvement, decreasing from 5.23 points before to 3.82 points after the intervention (*P* = 0.032, Table 4). In addition, the incidence rates of POI and infection decreased from 5.38% and 2.65% before to 1.67% and 0.84% after the intervention, respectively (*P* < 0.001, Table 4). Monthly data on patient rehabilitation before and after the intervention are summarized in Supplemental Table 1.

**Table 4:** Patient postoperative rehabilitation before and after the evidence-based intervention

Rehabilitation measures		Before the intervention (*n* = 226)	After the intervention (*n* = 239)	*P* value
Onset time of off-bed activities (hours after the operation, mean ± SD)		31.89 ± 9.50	20.01 ± 4.65	< 0.001
Off-bed rate within 24 h after operation, N (%)		69 (30.94)	218 (91.21)	< 0.001
Postoperative pain score in resting state		1.89 (0–5.38)	1.53 (0–3.89)	0.084
Postoperative pain score in active state		5.23 (1.76–8.34)	3.82 (2.16–5.12)	0.032
Incidence of postoperative pain score < 3 points, N (%)		168 (74.37)	200 (83.68)	0.014
Incidence of postoperative ileus, N (%)		12 (5.38)	4 (1.67)	< 0.001
Postoperative infection [11], N (%)	Total	6 (2.65)	2 (0.84)	< 0.001
	Superficial infection of incision	1 (0.44)	1 (0.42)	
	Deep infection of incision	2 (0.88)	0 (0.00)	
	Urinary tract infection	2 (0.88)	1 (0.42)	
	Pulmonary infection	4 (1.77)	1 (0.42)	
	bacteremia and other systemic infections	3 (1.33)	0 (0.00)	
Postoperative hospital stay (days, mean ± SD)		5.06 ± 1.99	3.51 ± 0.99	< 0.001

*SD* standard deviation

## Discussion

Our study showed that early mobilization after C-section conducted by a multidisciplinary health care team could singly shorten the time until the patient’s first ambulation, shorten the length of hospital stay and reduce complications after C-section. This result is consistent with those of many previous studies [18, 19], and early mobilization after C-section can improve patient recovery. In the study, all of the clinical practices included in the intervention were successfully implemented except abdominal drainage tube removal within 24 h and activity recording by patients, and the onset time of off-bed activities decreased from 31.89 ± 9.50 h to 20.01 ± 4.65 h.

Although the incidence of POI was reduced from 5.38% before to 1.67% after the intervention, as shown in the tables, both incidence rates were much higher for POI than in other research [20–23], which always had strict exclusion criteria, such as emergency operation, scarred uterus and other conditions that might influence gastrointestinal motility. In this study, the percentage of emergency operations was 47.35%, the scarred uterus complication was 45.85%, the duration of operation was 1.31 ± 0.63 h, and the blood loss was 382–714 ml (exceeding 500 ml was 74.78%) before the intervention, as shown in Table 2. According to previous study results, patients who had previous abdominal surgery, long duration of surgery, emergency surgery, blood loss exceeding 500 ml, extensive adhesiolysis, acute gestational complications such as preeclampsia and received magnesium sulfate, which may affect intestinal peristalsis, are at higher risk of developing POI [21, 22]. This is the reason why there is a high incidence of this serious complication both before and after the intervention in the clinical setting of the Guangzhou Obstetrics Critical Care Center in China.

Preoperative education and preoperative behavioural interventions have a direct impact on patients’ postoperative rehabilitation and were well documented in this study. According to previous study results, postoperative patients may suffer postoperative problems such as incision pain [19], intestinal distension [18], and incision site or urinary tract infections [19, 20], and they would refuse mobilization. Therefore, preoperative education of patients is an important part of postoperative care in C-sections [21]. In this study, preoperative maternal education was dominated by traditional oral education, and there was no implementation of a postoperative activity plan or daily activity goals. After the intervention, standardized preoperative health education was carried out in various forms, such as an activity manual with pictures and text, a propaganda column on the wall, and videos on TV in the ward. There was a significant increase in the rate of implementation of postoperative activities plans and daily activity goals that were communicated in writing by the nurse prior to the operation. It also relieved the worries of parturient women and their families so leaving bed for the first time after surgery occurred earlier, and the rate of leaving bed after 24 h was obviously improved. But the low compliance rate with activities recorded by patients and nurses with a ground scale was just 59.83%. The reasons were likely that it can not be recorded in real time and need to be manually added by the nurses, which increases the workload of the nurses.

In the present study, a shortened duration of indwelling drainage tube/catheter placement shortened the time to the patient's first ambulation and the hospital stay. After surgery, early mobilization and early ambulation are facilitated in most patients after the drainage tube is removed [22, 23]. Another study also found that complications of postoperative infection and POI tended to be higher in the drainage group than in the no-drainage group [23]. Abdominal drainage was first performed in abdominal surgery by Chassaignac in 1859 [24]. One of the main goals of prophylactic abdominal drainage placement at the end of the procedure was to shorten the time to diagnosis of haemorrhage [25]. Abdominal drain placement was generally considered a harmless preventive measure and still performed routinely [26] before the evidence-based intervention, so 15.49% of C-section patients received drainage before intervention. In an era where promotion and application of ERAS practices are gaining increasing acceptance, abdominal drainage is no longer undertaken routinely [10–14] and has decreased from 15.49% before to 7.11% (Table 3) after the intervention. However, this result is still slightly higher than findings in other research [4, 7]. There are two main reasons for this outcome: (1) Whether to perform abdominal cavity drainage after surgery because of the large wound, long operation time and extensive intraoperative bleeding in repeat caesareans is controversial [25]. (2) After a high risk of bleeding after surgery was established, such as intraoperative blood loss over 1000 ml, abdominal drainage is performed in China [26]. The removal of the drainage within 24 h remained quite low (41.17%), possibly because many patients who used a tube had severe pelvic adhesions and continuous build-up of fluid in the abdomen.

Previous studies[27] have shown that 71% of parturients are most worried about pain during early ambulation. The combination of local anaesthetics and opioids reduces the dose of opioids and has the benefit of achieving postoperative analgesia after C-section [28, 29]. This study showed that the localization analgesia programme standardized the postoperative analgesia administration procedure in the department and kept the postoperative pain score below 3, which also reduced the risk of POI due to improper use of opioids.

This study had several limitations. First, the clinical practices of this intervention were limited by the resources/technologies available in our Obstetrics Care Center. Second, this was a single-centre investigation. With the advance of modern information technology, application programs such as bracelets can be used to record patients’ activities in real time. Future multicentre trials employing more advanced technologies are required to further validate and improve the intervention.

## Conclusions

In conclusion, we established an evidence-based nursing intervention that can effectively promote early postoperative off-bed activities and rehabilitation among C-section patients in China. Multidisciplinary collaboration and multiple forms of preoperative health education for family members and patients could help promote evidence-based postoperative management, alleviate postoperative pain, shorten hospital stays, and decrease the incidence of POI and infection. Our study shows that evidence-based nursing intervention in C-section patients can significantly improve patient outcomes, and evaluation of the effect of evidence-based practices should be considered in the clinical setting.

## Supplementary Information

**Additional file 1: Supplemental Table 1.** Patient postoperative rehabilitation by month before and after the evidence-based intervention.

## Data Availability

The datasets used and/or analysed in this study are available from the corresponding author on reasonable request.

## Abbreviations

C-section: Caesarean section
ITS: Interrupted time series
ARIMA: Autoregressive integrated moving average
JBI: Joanna Briggs Institute
CSOG: Chinese Society of Obstetrics and Gynecology
CSS: Chinese Society of Surgery
FENCPG: Fudan Evidence-based Nursing Center Practice Guide
CNKI: China National Knowledge Internet
POI: Postoperative ileus
TAP: Transversus abdominis plane
SD: Standard deviation
R^2^: A goodness-of-fit measure for time series models
