# Supplementary material for: Embedding evidence of early postoperative off-bed activities and rehabilitation in a real clinical setting in China: an interrupted time-series study
Source: BMC Nurs. 2022 Apr 27;21:98. doi: 10.1186/s12912-022-00883-5 (PMC9044807; doi:10.1186/s12912-022-00883-5)
Supplement: Supplementary file 1 — Additional file 1: Supplemental Table 1. Patient postoperative rehabilitation by month before and after the evidence-based intervention. [file 12912_2022_883_MOESM1_ESM.docx]

**Supplemental Table 1. Patient postoperative rehabilitation by month before and after the evidence-based intervention.**

| Assessment | Before the intervention (n=226) | | | | | | After the intervention (n=239) | | | | | | P |
| --- | --- | --- | --- | --- | --- | --- | --- | --- | --- | --- | --- | --- | --- |
|  | 2019.2（n=42） | 2019.3（n=42） | 2019.4（n=45） | 2019.5（n=48） | 2019.6（n=44） | R^2^ | 2019.9（n=53） | 2019.10（n=46） | 2019.11（n=48） | 2019.12（n=48） | 2020.1（n=44） | R^2^ |  |
| Onset time of off-bed activities (hours after the operation) | 32.19 | 32.25 | 31.57 | 31.35 | 32.13 | 0.16 | 20.81 | 20.32 | 19.07 | 20.10 | 19.88 | 0.26 | <0.01 |
| Postoperative hospital stay (days) | 5.09 | 5.06 | 5.17 | 4.97 | 5.02 | 0.23 | 3.75 | 3.65 | 3.41 | 3.35 | 3.34 | 0.88 | <0.01 |
| Incidence of postoperative pain score < 3 points | 76.19% | 65.96% | 75.56% | 79.17% | 75.00% | 0.12 | 80.43% | 87.50% | 81.25% | 86.36% | 83.68% | 0.08 | <0.001 |
| Incidence of postoperative ileus | 4.80% | 8.50% | 4.40% | 4.17% | 4.50% | 0.18 | 3.80% | 2.17% | 0.00% | 0.00% | 2.27% | 0.26 | 0.03 |
| Incidence of postoperative infection | 0.06% | 0.05% | 0.05% | 0.04% | 0.10% | 0.34 | 0.04% | 0.05% | 0.00% | 0.00% | 0.00% | 0.66 | 0.01 |
